# Topology-aware design of spiking neural networks via modular graph architectures

**DOI:** 10.1371/journal.pone.0344997

**Published:** 2026-04-10

**Authors:** Farideh Motaghian, Soheila Nazari, Juan P. Dominguez-Morales, Reza Jafari

**Affiliations:** 1 Physics Department, Shahid Beheshti University, Tehran, Iran; 2 Escuela Politecnica Superior, Universidad de Sevilla, Sevilla, Spain; 3 Faculty of Electrical Engineering, Shahid Beheshti University, Tehran, Iran; 4 Robotics and Technology of Computers Lab., ETSII-EPS, SCORE Lab, Universidad de Sevilla, Sevilla, Spain; 5 Chandigarh Group of Colleges Jhanjeri, Sahibzada Ajit Singh Nagar, Panjab, India; Georgia State University, UNITED STATES OF AMERICA

## Abstract

Spiking Neural Networks (SNNs) offer a biologically plausible and energy-efficient alternative to traditional artificial neural networks (ANNs), yet their design remains constrained by limited architectural flexibility and slow training dynamics. In this work, we introduce a novel SNN framework that leverages modular graph-based topologies and explicit synaptic delays to significantly enhance both training efficiency and classification performance. Our architecture, TANet-Tiny, incorporates structured graph stages with up to 32 nodes and diverse community-driven connectivity patterns derived from KMeans clustering, Louvain modularity, and Watts–Strogatz small-world models. We integrate these topologies into a topology-aware search space and explore them via a Spatio-Temporal Topology Sampling (STTS) approach, enabling the discovery of high-performing networks without exhaustive search. Experimental results on MNIST, CIFAR-10, and CIFAR-100 demonstrate that our modular designs achieve state-of-the-art accuracy while requiring 6–10 × fewer training epochs, with top-1 accuracy reaching 99.57% on MNIST and over 92% on CIFAR-10, all with reduced parameter counts. We introduce an accuracy-per-epoch metric to quantify training efficiency and show that modularity, rather than network size, is the critical driver of performance. This work lays the groundwork for scalable, interpretable, and low-latency SNN architectures suitable for deployment in neuromorphic and edge computing environments.

## 1. Introduction

Spiking Neural Networks (SNNs), are frequently regarded as the third generation of neural network architecture, hold enormous promise for facilitating artificial intelligence, particularly in energy-efficient computation and spatio-temporal data processing. Inspired by the temporal coding and event-driven communication of the brain, SNNs offer an ideal substitute for traditional Artificial Neural Networks (ANNs) [1]. As opposed to ANNs, which employ continuous values and synchronous processing, SNNs communicate with discrete events (spikes) using the precision of spike timing for computation. Plasticity at synapses, typically achieved by Spike-Timing Dependent Plasticity (STDP), is the key learning mechanism of SNNs, where the timing relationship between post- and pre-synaptic spikes dictates synaptic weight adjustments.

Despite their potential, designing successful SNN architectures that fully utilize their inherent capabilities is still an uphill task. Multimodal adaptation of renowned ANN architectures like VGG-Net [2] and ResNet [3] to the spiking domain has been successful to some extent but at the cost of de-emphasizing the fundamental distinctions between the two paradigms at the expense of potentially compromising performance and energy efficiency on neuromorphic chips. The very spatio-temporal nature of SNNs mandates architectural design aspects beyond the narrowly feedforward architectures so prevalent in many ANNs.

One of the most crucial factors influencing SNN performance and biological plausibility is network connectivity, or topology. Connectivity between neurons determines how information flows and is processed in the network. Modularity is very high in biological neural systems with various brain regions having specialized functions and being connected by sparse connections [4,5]. This biological inspiration has driven work on modular structures in SNNs, with neurons being grouped into functional assemblies [6]. Some of the early SNN research explored fixed connection patterns like small-world networks [7], as first steps towards explaining some aspects of biological structure and sacrificing local processing for the sake of global communication. More recent work recognizes that flexible and optimized connection topologies are central to designing complex and efficient modular SNNs [8,9].

Several recent studies have explored other network topologies of SNN design. For instance, Yan et al. [10] proposed sampling complex topologies using random graphs and achieved performance enhancement. Other studies used models like Barabási–Albert (BA) and Watts–Strogatz (WS) to model real-world network topology [References]. However, a key limitation of such studies is that they typically do not directly enforce community structures or modularity to control learning and computation in the network.

In this work, we introduce a novel graph-driven architectural paradigm for spiking neural networks that explicitly integrates community detection concepts from complex network theory.

Instead of relying on fixed or implicitly modular topologies, the proposed approach constructs SNN connectivity graphs with explicitly detected communities and sparse inter-community connections, directly embedding modular organization into the network architecture.

This explicit modularity enables localized learning and structured information flow, resulting in reduced energy consumption, faster training convergence, and improved classification performance compared to non-modular and randomly connected SNN architectures.

On the other hand, this work introduces and investigates formally the impact of modularity in SNN network architecture by creating graphs with distinct communities and sparse inter-community connections. This method draws inspiration from speculation that overt modularity may enable localized learning, enhance feature specialization within modules, and perhaps reduce overfitting, capturing principles observed in biological neural systems.

Discovering the optimal SNN architectures, particularly those with complex and dynamic topologies, is a computationally challenging problem. Classic neural architecture search (NAS) approaches, extensively used for ANNs, are greatly hindered when the reduced speed of training of spiking models [10,11] is taken into account. Existing NAS methods proposed for SNNs [12–14] have attempted to mitigate this by limiting the search space, typically restricting networks to their small cell-based versions. While this accelerates the search process, it also significantly constrains the diversity of topologies explored, potentially missing highly effective SNN designs.

To remove these constraints and allow for more diverse and high-performance SNN structures to be discovered, we present a topology-aware SNN search space that is able to support a more extensive spatial and temporal network architecture. Our search space significantly enhances the graph size, with a support of 32 nodes — eight times higher than previous work [12–14] — and includes synaptic delays explicitly to represent temporal dynamics. This novel technique enables enhanced utilization of the native temporal processing capabilities of SNNs. With the substantial increase in this search space, the Spatio-Temporal Topology Sampling (STTS) model is introduced. Following the competitive performance of random search in some NAS environments [15–17], STTS leverages random graph models and samples synaptic delays to search through this immense space in an efficient manner and find competitive architectures in seconds, a significant acceleration compared to conventional SNN NAS approaches.

By comparing classification performance systematically across graph topologies, including those with explicit modularity, and introducing an efficient mechanism to search complex topologies, we aim to demystify how network organization impacts the learning capabilities of SNNs. This work informs the design of more biologically plausible, energy-efficient, and high-performance SNN architectures.

The main contributions of this paper include the following:

- Novel spiking neural network designs are proposed, which explicitly incorporate modularity and community structures to enhance training efficiency and generalization.
- Three modular graph generation methods (Watts–Strogatz small-world networks, Louvain-based modular graphs, and K-means-based modular graphs) are introduced to systematically explore the impact of topological structures on SNN performance
- The proposed architectures demonstrate competitive classification accuracy on MNIST, CIFAR-10, and CIFAR-100 with significantly faster convergence (fewer training epochs) compared to the state of the art.

The structure of this paper is outlined as follows. Section 2 offers a detailed explanation of the proposed methodology, i.e., SNN architecture, graph generation techniques, datasets, and training algorithms. Section 3 outlines experimental results concerning the performance comparison of different graph topologies and the performance of the STTS algorithm. Section 4 discusses implications of the results and concludes the research. Section 5 outlines directions for future work, including theoretical analysis of the role of topology in SNN performance.

## 2. Methodology

This section describes the proposed methodology, i.e., evaluation datasets, Spiking Neural Network structure, graph structure generation strategies, and training strategies.

### 2.1 Datasets

In an attempt to confirm the effectiveness and performance of our proposed SNN structure under various graph generation strategies, we tested three most widely utilized image classification datasets: MNIST, CIFAR-10, and CIFAR-100. These datasets cover a variety of complexity levels with respect to image resolution, number of classes, and inter-class variability, allowing for exhaustive testing of the model’s scalability and robustness. Datasets were processed in a way consistent with practices described in the literature. The MNIST dataset includes 70,000 gray-scale handwritten digit images (0–9) with 60,000 images as train data and 10,000 images as test data. Each image is of size 28 × 28 pixels. It is a common benchmark to evaluate image classification models [18]. The CIFAR-10 dataset includes 60,000 32 × 32 RGB images of 10 object classes like cat, airplane, and automobile with 50,000 images as train data and 10,000 images as test data [19]. CIFAR-100 dataset is of the same setup as CIFAR-10 albeit with 100 classes and 600 images in each class. There is a hierarchical labeling system, but we have only made use of fine labels for the classification. Training and test splits remain at 50,000 and 10,000, in respective order [20].

For all the datasets, regular data augmentation techniques (e.g., random crops, horizontal flip), normalization and PCA were performed at training time to improve generalization performance, as is standard practice when training deep neural networks on these datasets. Input pixel values were typically normalized to some range or z-scored. Spike trains were generated by transforming the input images, for example, into some rate coding or latency coding.

### 2.2 Spiking neural network architecture

Overall Structure: The proposed Spiking Neural Network (SNN) architecture employs a stage-wise structure, borrowing from contemporary deep network structures. The modular architecture facilitates the construction of a deep SNN by stacking several stages of computation, a common deep learning practice [1,21]. Our structure specifically consists of features analogous to successful deep architectures, namely the stage-wise and graph-based structure presented in the TANet-Tiny work [9].

#### 2.2.1 Stages and architecture design.

**Fig 1:**
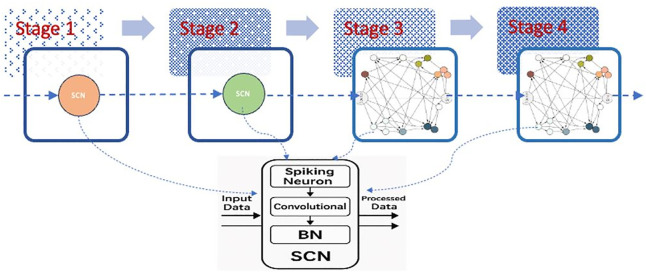
Stages include graph architecture.

**Fig 1** depicts the general multi-stage design of the proposed overall TANet architecture. Each of the individual stages in such a process is here represented as a graph-based module where nodes correspond to basic computational units and edges define the structured synaptic connectivity. The stages are then cascaded, with the sink node of each stage acting as the source node for the next stage, allowing for hierarchical feature extraction and progressive spatial resolution reduction across the network.

Specifics on the configuration of the TANet-Tiny architecture used in this work are provided in Table 1. For each stage, the table names the type of structure, number of nodes in the connectivity graph, and the dimensions of the output channel. As can be seen, the first two stages have simple single-node structures, while the deeper stages adopt DAG topologies with 32 nodes, allowing for richer modular computation and topology-aware information flow in higher-level feature representations.

**Table 1:** The architecture of TANet-Tiny is defined by its stages, each of which is produced from a connection topology graph. Within each stage’s graph, *N* specifies the quantity of nodes and C representing the output channel count.

Stage	Structure Type	Number of Nodes	Output Channels
Stage 1	Single Node	N = 1	C = 96
Stage 2	Single Node	N = 1	C = 96
Stage 3	Graph (DAG)	N = 32	2C = 192
Stage 4	Graph (DAG)	N = 32	4C = 384
Classifier	Fully Connected Layer		Number of Classes

#### 2.2.2 Node representation.

**Fig 2:**
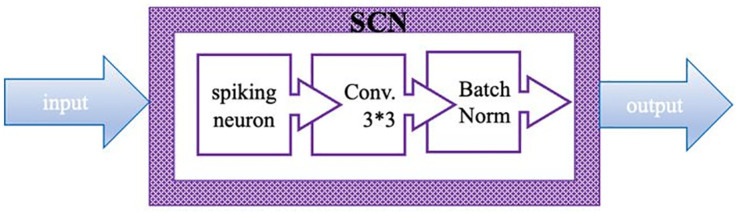
Represent Spiking Convolution Node (SCN) include of Spike neuron, convolution 3*3 layer and batch normalization layer.

For all graph stages, each node is projected onto a Spiking Convolution Node (SCN). SCN is the fundamental computation component of the graph topology. From **Fig 2**, an SCN accepts input data from its input edges and outputs processed data through its output edges. In an SCN, the computation process happens in some order: first, it accumulates all incoming spiking data through simple summation. This pooled data is subsequently processed in a sequential manner by a spiking neuron (SN) layer, a convolutional layer, and finally a batch normalization (BN) layer. This specific order of processing (SN-convolution-BN) is shared by all SCNs in the graph stages and is not part of the topology search space. This combination allows all the nodes to perform both spatial feature extraction through convolution and temporal processing based on the spiking neuron dynamics [23,24]. During inference, the parameters of the Batch Normalization layer are added into the earlier convolutional layer for computational efficiency. The individual options of the convolutional layer for each SCN (e.g., number of output channels, stride, kernel size, padding) can vary according to the node’s position within the graph and are part of the Layer Details.

#### 2.2.3 Edge representation.

The directed edges of the graph represent the synaptic connections among the Spiking Convolution Nodes (SCNs). These connections are spike-based, i.e., information is transferred as a sequence of discrete events (spikes) between the connected nodes [1]. Apart from the spatial topology of connection between nodes specified by which nodes are connected, our architecture also considers the temporal topology of connections. This temporal aspect is controlled by the synaptic delay parameter of each edge. Synaptic delay, or the time it takes for a spike to travel across a synapse, has been shown to heavily influence the efficiency and effectiveness of SNNs by regulating the passage and processing of temporal information [1].

Unlike most of the prior SNN work that does not consider synaptic delay, we actually do consider it in our topology design specifically. In our graph-based architecture, each edge contains an additional parameter controlling its synaptic delay. For simplicity of implementation, we restrict these synaptic delays to a set of discrete values. This allows the specification of a temporal topology as well as a spatial one, so that the network can utilize the high temporal dynamics that exist in spiking signals.

It is noteworthy that the topological ordering and directed edges of nodes in every graph stage ensure strictly feedforward information flow at stage level, with no recurrent connections. Such feedforward structure simplifies training and analysis compared to recurrent SNNs. Directed connections and topological ordering of computational blocks are also concepts in efficiency-driven architectures [25].

#### 2.2.4 Layer specifications.

**Fig 3:**
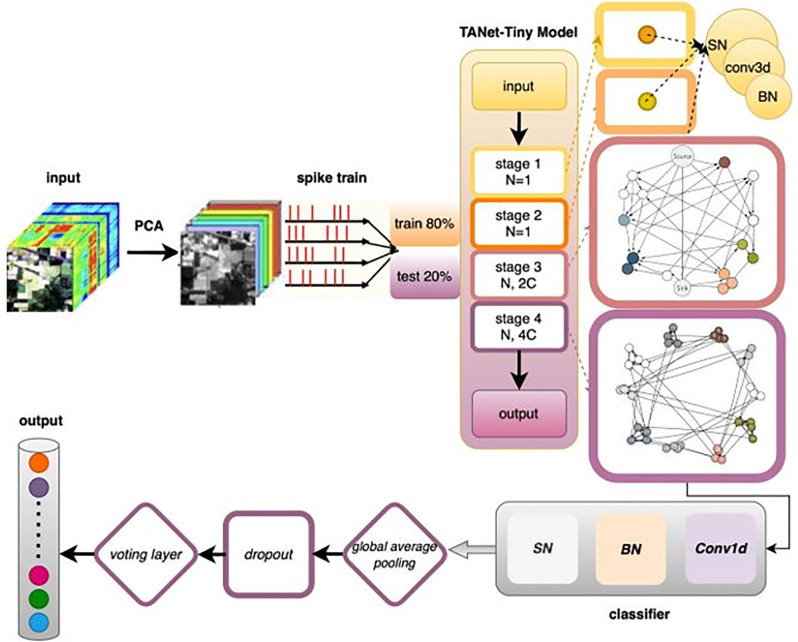
Schematic of deep SNN with modular graph topology.

The real specification for the type of layer in the network is critical to its performance. In the first 2D convolutional layer, we will assign the kernel size (e.g., 3 \times 3), number of output filters (e.g., 64), stride (e.g., 1), and padding (e.g., ‘same’). Similarly, the potential sizes for the spiking convolution nodes across the graph stages will be defined in the search space as the potential size range for kernel sizes, filter numbers, stride, and padding. If the graph convolution layers are used, their respective aggregation functions, numbers of output feature, and other relevant hyperparameters will be added. The linear classifier on the output stage will have input size identical to that of the Global Average Pooling layer’s output and output size identical to the classes in the classification task. Fig 3 illustrates the schematic representation of a deep spiking neural network (SNN) featuring a modular graph topology, highlighting the organization and interconnectivity of the network’s modular components.

#### 2.2.5 Spiking neuron model.

The Leaky Integrate-and-Fire (LIF) neuron model serves as the core computational component of our SNN [7]. The following differential equation for the membrane potential of the LIF neuron describes its behavior *V*_*m*_*(t)*:


$$\tau m\frac{dVm(t)}{dt}=(Vm(t)-V_{rest})+RmI(t)$$ (1)

where *τ*_*m*_ denotes the membrane time constant, *V*_*rest*_ represents the resting membrane potential, Rm indicates the membrane resistance, and *I(t)* signifies the input current. When the membrane potential *V*_*m*_*(t)* attains a threshold *V*_*th*_, the neuron generates a spike, and its membrane potential is subsequently reset to a reset potential *V*_*rest*_ for a refractory duration *τ*_*ref*_. The input current *I*(*t*) to a spiking neuron is typically the sum of weighted incoming spikes from presynaptic neurons. This model captures essential dynamics of biological neurons while being computationally efficient [1]. Recent work has also explored using membrane time constants that can be learnt to improve learning in SNNs [23].

#### 2.2.6 Synaptic delays.

Synaptic delays are crucial in defining the temporal behavior of spiking neural networks (SNNs). In our design, synaptic delays are enforced on the edges connecting spiking convolution nodes across different graph stages. Each directed edge in the graph is associated with a specific delay value, denoted as $d_{ij},$ which can be influenced by a delay parameter p. When a presynaptic neuron $N_{i}$ emits a spike at time t, the spike will reach the postsynaptic neuron $N_{j}$ after a delay dependent on


dij=p·Δt (2)

Where Δt is a scale factor relevant to the network dynamics. Thus, the arrival time of the spike at the postsynaptic neuron can be expressed as:


$$t^{\prime }=t+dij=t+p\cdot \Delta t$$ (3)

This synaptic delay introduces a temporal factor into the information processing at each level of the network, allowing temporal dependencies to be learnt by the SNN in the input data.

The scope and range of these synaptic delays will be defined within the architectural search space. By implementing these delays, we maintain the topological ordering of the network, allowing for feedforward spike propagation with temporal gaps. The impact of the synaptic delay parameter p on network performance is carefully evaluated, and based on our experiments, an optimal value of p=0.05 was determined for the datasets considered.

### 2.3 Graph generation strategies

In this section, we discuss the algorithms used to generate different graph topologies for our experiments. Our previous success in achieving high accuracy with modular graphs in neural network architectures provided the impetus to investigate their potential application within deep spiking neural networks. Consequently, we explored various modular graph configurations in this context [26]. The employment of a modular structure in the spiking neural network (SNN) architecture is important in promoting performance and pattern discernibility. Modularity enables dedicated functionality in independent modules, hence promoting specialization and effective information processing. Such a setup mirrors the sophistication of biological systems, which tend to demonstrate specialized functions alongside strong communication. By exploring different connectivity schemes across the graph stages, our interest is in observing what we can discover about the influence of such topological formations on the learning dynamics of the Spiking Neural Network (SNN) model. Every graph constructed outlines the connection pattern (edges) of the spiking convolution nodes (vertices) within a particular stage and thereby facilitates observation of how structural change affects overall performance and responsiveness.

#### 2.3.1 General parameters.

All graph generation methods have parameters that are held constant or within a pre-specified range to ensure a similar comparison and control the scale of the graphs throughout every stage. The total number of nodes per stage is one such parameter, which controls the size of the computational block. Similarly, the average degree (number of edges in each node) or the total sum of all edges is controlled in order to manage network density and computational cost. These macro parameters will be specified as part of the overall architectural search space.

#### 2.3.2 Modular graphs.

The overall objective of generating modular graphs is to generate structures that consist of clearly defined communities or groups of nodes well linked within themselves while poorly linked between themselves. This mimics some of the organizational structures present in biological neural networks and can facilitate specialist processing within neighborhoods and supervised information exchange between neighborhoods. All modular graphs in this work are constructed to have up to 10 neighborhoods. Sparse connectivity between the 10 neighborhoods is one control parameter. This is typically achieved by assigning a lower probability or a fixed, small number of edges between nodes across communities compared to nodes within the same community. This parameter directly influences the level of interaction and information exchange between the processing modules bounded by the communities. Within-community connections are generated to be comparatively dense. This promotes high-level interactions and possibly rapid information diffusion among nodes of the same community. The approach to forming these internal links (e.g., random links with high probability, or special patterns like small-world forms) depends on the specific modular graph generation algorithm used. Graph Generation Strategies for SNN Stages

In essence, the connectivity within the graph stages is not fixed but is defined by specially generated sparse graph structures.

This allows us to explore the impact of different network topologies on the learning behavior and computational efficiency of the SNN. Unlike employing the Barabási–Albert (BA) model [6] to produce scale-free graphs as has been done in some previous work, we explore three alternative graph generation methods for producing networks with controlled modularity and small-world properties: K-means-based modular graphs, Louvain-based modular graphs, and Watts–Strogatz small-world networks (SW) [5]. The primary motivation for employing such organized topologies is to facilitate more efficient information flow and possibly reduced convergence time during training, and with fewer epochs than in less organized or extremely dense networks. To study the impact of modularity and sparseness on the learning dynamics and computational efficiency of spiking neural networks (SNNs), we constructed synthetic directed graphs with very controlled topologies.

All graphs contained N=32 nodes, the neurons number at stage 3 and 4 in a network. In previous NAS-based SNN studies, the search space was highly constrained, with each cell typically containing only four nodes, limiting topological diversity [12,13]. Yan et al. (2024) [9] expanded this design to architectures with up to 32 nodes, enabling richer spatial and temporal connectivity patterns. Following this rationale, we evaluated larger configurations, including 64-node graphs. However, increasing the number of nodes beyond 32 did not yield accuracy improvements and in some cases led to slight degradation, likely due to reduced channel capacity under a fixed computational budget and increased structural complexity. Therefore, TANet-Tiny was configured with 32 nodes, which provides a balanced trade-off between topological richness, community-aware modularity, computational efficiency, and stable performance.

An entirely connected directed graph containing so many nodes would contain 2×(N2) is equal 992 possible directed edges. For our computations, however, we specifically restricted each graph to some 200 directed edges. This sparsification was essential, as it enabled modular organization to develop with sufficient connectivity to allow for useful information transfer. Most importantly, the patterns of connectivity within each graph were not fixed but instead generated by using structured sparse graph construction techniques. Rather than using traditional random or scale-free models such as Barabási–Albert (BA), we used three distinct graph generation strategies that promote modularity and small-world properties.

To provide further clarity on the role of modular graphs within our SNN architecture, we define modularity as the deliberate organization of neurons into densely connected subgroups (communities) characterized by comparatively sparse cross-community connections. For each graph (N = 32 nodes, approximately 200 directed edges), the network is partitioned into a fixed number of communities. Edges within the same community are generated with a significantly higher probability than those connecting different communities, resulting in graphs consistently exhibiting high modularity values (e.g., a Modularity of approximately 0.8).

From a neural computation perspective, each community functions as a localized processing unit, specialized in feature extraction. The dense intra-community connectivity facilitates rapid spike propagation and robust local integration of information. Conversely, the sparse inter-community links serve as controlled communication channels, regulating information flow between these specialized processing modules. This architectural design minimizes redundant global interactions, curtails unnecessary spike transmission, and promotes a highly structured, efficient information flow across the network’s processing stages.

Crucially, the underlying graph topology, including its modular organization, is established prior to training and remains fixed throughout the learning process. Only synaptic weights are optimized during training. This methodology allows us to rigorously isolate the functional contribution of structural modularity from the effects of synaptic plasticity. Empirically, we observed that organizing neurons into 10 communities provided superior alignment with the inherent class-level structures present in datasets such as MNIST and CIFAR-10, leading to improved accuracy and more stable convergence.

The resolution at which modular structures are defined plays a pivotal functional role in the network’s performance. In our 32-node directed graphs, this resolution is directly controlled by the number of communities. Through extensive empirical evaluation with various partition configurations, we determined that a 10-community organization yielded the best overall performance, particularly for MNIST and CIFAR-10. A finer resolution (i.e., a greater number of communities) resulted in excessive fragmentation and hindered effective global coordination, whereas a coarser resolution (fewer communities) diminished specialization by enlarging the processing modules. The chosen 10-community configuration thus represents an optimal balance between localized spike integration and efficient inter-community communication. These findings strongly suggest that modular resolution is not merely a structural parameter but an intrinsically interlinked feature of functionality, profoundly shaping information propagation dynamics, convergence behavior, and ultimately, the network’s classification performance.

**2.3.2.1** **Watts–Strogatz Small-World Networks**

To model graphs with small-world properties, we adapted the Watts–Strogatz model to the directed domain. Starting with a directed ring lattice where each node was connected to its *k* nearest neighbors, we randomly rewired a small fraction p of edges. This introduced “shortcuts” while preserving high local clustering.

The small-world property arises when a network has both short average path length and a high clustering coefficient. Defining the clustering coefficient in directed networks requires extra care in how directionality of edges is treated [27]. There are many different definitions, and we followed a procedure to measure the probability that two nodes connected directly to a central node *i* are also connected to one another, with consideration of edge direction.

The region clustering coefficient C*ᵢ* of a node *i* is given by:


$$C_{i}=\frac{\text{Number of existing directed triplets involving node }i\text{that are closed}}{\text{Maximum possible number of directed triplets involving node }i\text{that could be closed}}$$ (4)

This definition generally expresses the ratio of existing closed triplets (cycles of length three) involving node *ii* to the total number of potential closed triplets around that node. The global clustering coefficient *C* is the average of *Ci* over all nodes in the network. The global coefficient Cis the average over all nodes, *k*_i_ is sum of input and output degrees. A graph is considered small-world if C *≫* $C_{random}$*,* where $C_{random}$ is the expected clustering of a random directed graph with similar degree distribution.

Although not explicitly designed for modularity, high clustering in small-world networks will generate emergent community-like subgraphs [28]. These subgraphs possess dense local connections and loose connections to the remainder of the network. This combination of dense local connection and global shortcuts allows rapid spreading of information, rapid signal transfer, and enhanced robustness to random failure—key properties found in many real networks, from biological neural networks to social networks of communication.

The clustering coefficient in small-world networks is found to vary in the range of 0.1 to 0.4. These values are significantly higher compared to random networks, which typically lie below 0.01. Small-world networks, on the other hand, have exceptionally short average path lengths that require just a few steps. These average path lengths lie in the range of *log N/ log < k>* (where N is the number of nodes and k is the degree of each node) and vary from 2 to 6 [7].

**2.3.2.2** **Louvain-Based Modular Graphs**

To create modularity rooted in community detection, we drew inspiration from the Louvain algorithm, which identifies communities by maximizing modularity—a measure of the density of intra-community links compared to a null model. The modularity Q is described as:


$$Q=(\text{1}/\text{2}m)\Sigma_{\text{ij}}[A_{\text{ij}}-(k_{\text{i}}k_{\text{j}}/\text{2}m)]\delta (c_{\text{i}},c_{\text{j}})$$ (5)

where A_ij_ is the adjacency matrix, *k*_i_ and *k*_j_ are node degrees, *m* is the total number of edges, and $\delta \left(c_{i},c_{j}\right)=\text{1}$ if nodes i and j belong to the one community. We began by manually labeling nodes into communities in a weakly connected directed graph, assigning intra-community edges with higher probability than inter-community ones. The Louvain method was then applied (to the undirected version of the graph) to refine and sharpen community boundaries. This two-stage process ensured both intuitive control over community labels and algorithmic optimization of modularity. The graph was further tuned to maintain a consistent target edge count (~200 directed edges).

2.3.2.3 K-means-Based Modular Graphs

This approach utilizes the algorithm of K-means clustering to impose modular structure on a graph. Each node is initially assigned synthetic coordinates in a *2D* feature space. The K-means algorithm partitions nodes into k clusters by minimizing intra-cluster variance, formally defined by the objective:


$$J={\Sigma_{i=\text{1}}}^{\text{k}}\Sigma_{\text{x }}\varepsilon {\text{C}}_{\text{i}}\;{∥\;x-\mu_{i}\;∥}^{\text{2}}$$ (6)

where *C*_i_ is the set of nodes in cluster *i*, and $\mu_{i}$ is the centroid of that cluster. The resulting clusters serve as modules for the graph. Directed intra-cluster edges are densely formed, while inter-cluster edges are added more sparsely, producing visually and structurally distinct communities. This synthetic modularity supports localized processing within clusters, with regulated information flow between them. Graph modules are directly controlled by the number of clusters, k.

**Fig 4:**
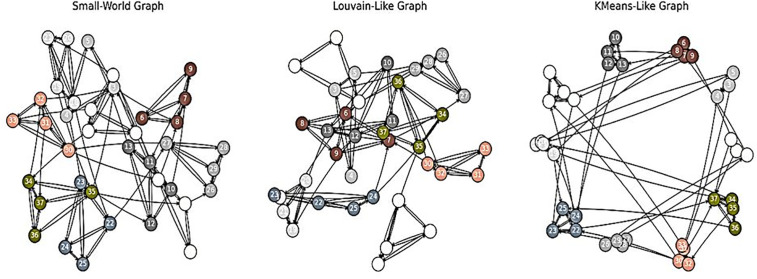
Modular graphs: small-world topologies, Louvain community detection and Kmeans clustering.

These diverse approaches yield graph datasets with distinct clustering patterns, community structures, and connectivity distributions. As illustrated in **Fig 4**, the generated graphs span a range of modular and small-world topologies, offering rich ground for investigating the relationship between graph structure and learning dynamics.

### 2.4 Training process

The architected SNN model, with the new graph generation methods in its stages, was end-to-end trained using Spatio-Temporal Backpropagation (STBP). STBP is a widely used training technique for SNNs that addresses the issue of gradients backpropagation through the non-differentiable spiking activity of the neurons [29]. It achieves this by using surrogate gradients at the spike points in such a way that gradient-based optimization techniques widely employed in Artificial Neural Networks (ANNs) can be utilized.

For the task considered in this paper, the following loss function was utilized:


$$Loss=-\sum_{K}y_{K}\text{log}\left(P_{K}\right)+\gamma \sum_{i\notin K}\sum_{t}{\left(\overset{t}{\underset{i}{Spike}}\right)}^{\text{2}}$$ (7)

$$P_{K}=softmax\left(\sum_{t}\overset{t}{\underset{K}{Spike}}\right)=\frac{\prod_{t}e^{\overset{t}{\underset{c}{Spike}}}}{\sum_{i\epsilon K}\prod_{t}e^{\overset{t}{\underset{i}{Spike}}}}$$ (8)

The label is denoted by $y_{K}$. K indicate the predicted neuron. γ represents the coefficient for regularization. It does not influence the classified neurons K. The probability of prediction, denoted as $P_{K}$, is determined using the softmax function. If the simulation time during the operation of the SNN is considered as T, the following is the formalization of the derivative of the loss function with regard to the spike time $\overset{t}{\underset{K}{Spike}}$:


$$\frac{\partial Loss}{\partial \overset{t}{\underset{K}{Spike}}}=P_{K}-y_{K}$$ (9)

Given that $\overset{t+\text{1}}{\underset{K}{S}}$ is dependent on $\overset{t}{\underset{K}{S}}$, derivative of the loss function with considering other neurons can be express as follows when t<T.


$$\frac{\partial Loss}{\partial \overset{t}{\underset{K}{Spike}}}=P_{K}-y_{K}+\sum_{i}\frac{\partial Loss}{\partial \overset{t+\text{1}}{\underset{i}{Spike}}}\times \frac{\partial \overset{t+\text{1}}{\underset{i}{Spike}}}{\partial \overset{t}{\underset{K}{Spike}}}$$ (10)

It should be noted that neurons j∉K do not receive any backpropagated error when t equals T and if t<T, the error of neuron j∉K can be calculated from the following equation.


$$\frac{\partial Loss}{\partial \overset{t}{\underset{j}{Spike}}}=\sum_{i}\frac{\partial Loss}{\partial \overset{t+\text{1}}{\underset{i}{Spike}}}\times \frac{\partial \overset{t+\text{1}}{\underset{i}{Spike}}}{\partial \overset{t}{\underset{j}{Spike}}}+\gamma \overset{t}{\underset{j}{Spike}}$$ (11)

In summary, the backpropagation error for all neurons and times is summarized in the following relationship:


$$\frac{\partial Loos}{\partial \overset{t}{\underset{j}{Spike}}}=\left\{ \begin{array}{c} @cP_{j}-y_{j}t=T,j\epsilon K\\ \gamma \overset{t}{\underset{j}{Spike}}t=T,j\notin K\\ P_{j}-y_{j}+\sum_{i}\frac{\partial Loos}{\partial \overset{t+\text{1}}{\underset{i}{Spike}}}\times \frac{\partial \overset{t+\text{1}}{\underset{i}{Spike}}}{\partial \overset{t}{\underset{j}{Spike}}}t<T,j\epsilon K\\ \sum_{i}\frac{\partial Loos}{\partial \overset{t+\text{1}}{\underset{i}{Spike}}}\times \frac{\partial \overset{t+\text{1}}{\underset{i}{Spike}}}{\partial \overset{t}{\underset{j}{Spike}}}+\gamma \overset{t}{\underset{j}{Spike}}t=T,j\notin K \end{array}\right.$$ (12)

Finally, the weights and bias can be updated as follows:


$$\frac{\partial Loss}{\partial w_{i,j}}=\sum_{t}\frac{\partial Loss}{\partial \overset{t}{\underset{i}{Spike}}}\times \frac{\partial \overset{t}{\underset{i}{Spike}}}{\partial w_{i,j}}$$ (13)

$$\frac{\partial Loss}{\partial b_{i}}=\sum_{t}\frac{\partial Loss}{\partial \overset{t}{\underset{i}{Spike}}}\times \frac{\partial \overset{t}{\underset{i}{Spike}}}{\partial b_{i}}$$ (14)

#### 2.4.1 Optimization.

We used the Stochastic Gradient Descent (SGD) optimizer for training with momentum 0.9 and weight decay 5e-4. The cosine annealing schedule was used for learning rate scheduling. The learning rate began at 0.1 and annealed to 0 over training.

Number of Time Steps: Training and all SNN simulations were performed with a constant number of time steps, specifically 4. This number was constant for all datasets and graph structures to ensure a consistent evaluation framework and to disentangle the impact of the graph structures on performance and efficiency.

#### 2.4.2 Batch size.

Training was performed with a batch size of 96 samples.

#### 2.4.3 Data augmentation.

For every dataset, we applied standard data augmentation to the training set in order to improve the model’s ability for generalisation and avoid overfitting. The augmentations depended on the character of each dataset for MNIST since the images are relatively straightforward, we employed random cropping with padding 4 pixels and a 0.5-probability random horizontal flip. For CIFAR-10 and CIFAR-100, more complicated datasets, we used a blend of random cropping padded with 4 pixels, random flip horizontally with probability 0.5, and Cutout (DeVries & Taylor, 2017). Cutout was used by masking the square region of 16x16 pixels from the images. These augmentation methods are routine and were found to be effective in training deep learning models on these datasets with techniques like those in [9].

#### 2.4.4 Normalization.

All input images of the datasets were normalized channel-wise according to training set mean and standard deviation. The following specific normalization parameters have been utilized:

- MNIST: Mean: 0.1307, Standard Deviation: 0.3081
- CIFAR-10: Mean: (0.4914, 0.4822, 0.4465), Standard Deviation: (0.2023, 0.1994, 0.2010)
- CIFAR-100: Mean: (0.5071, 0.4865, 0.4409), Standard Deviation: (0.2673, 0.2564, 0.2761)

#### 2.4.5 Batch normalization.

In order to enhance the convergence and stability of training, Batch Normalization [22] layers were strategically inserted into the network architecture. Specifically, prior to the activation function and following each convolutional layer, batch normalization was used. (spiking neuron model). Higher learning rates are made possible and internal covariate shift decrease as a result. Given that the complete whitening of the inputs for each layer is expensive and not universally differentiable, we implement two essential simplifications. The first simplification involves normalizing each scalar feature independently, rather than jointly whitening the features in the inputs and outputs of the layer. This normalization will ensure that the variance of each feature is one and its mean is zero. For a layer with a d-dimensional input represented as x=(x(1)...x(d)), we will proceed to normalize each dimension as follows:


$${\hat{x}}^{\left(k\right)}=\frac{x^{\left(k\right)}-E[x^{\left(k\right)}]}{\sqrt{Var[x^{\left(k\right)}]}}$$ (15)

where the $E[x^{\left(k\right)}]$ and $Var[x^{\left(k\right)}]$ are calculated based on the training process. As demonstrated in [30], this normalization accelerates convergence, even in the uncorrelated features. It is important to recognize that merely normalizing each input of a layer can alter the representations that the layer is capable of. For example, normalizing the inputs of a sigmoid would delete the nonlinearity. To overcome this problem, we used $\gamma^{\left(k\right)}$ and $\beta^{\left(k\right)}$ for each x(k), which serve to make scalability and shift to the normalized value and computed during training process:


$$y^{\left(k\right)}={\hat{x}}^{\left(k\right)}\gamma^{\left(k\right)}+\beta^{\left(k\right)}$$ (16)

#### 2.4.6 Hardware and software.

Experiments were conducted on an NVIDIA A100 GPU. SNN architecture and training process were implemented using the PyTorch deep learning framework with the parameter described in Table 2.

**Table 2:** Parameters and hyperparameters for training on MNIST, CIFAR-10/100.

Category	Parameter	Value	Description
**SpikingJelly**	T	4	Number of time steps for SNN simulation
	Tau (τ)	2.0 ms	Membrane time constant in LIF neuron
	V_threshold_	1.0 v	Voltage threshold at which the neuron fires
	V_reset_	0.0	Voltage to reset to after firing
	surrogate_function	Sigmoid ()	Function used for backpropagation through spikes
	graph-model(N = 32, Edge = 200, N.community=<10)	SW, Louvain, Kmeans	Type of graph used in model architecture
	skip-ratio	0.05	Edge-skipping rate or dropout-like mechanism
	batch-size	96	Batch size for training
**Training Hyperparameters**	Dataset	MNIST/ CIFAR-10/ CIFAR-100	Dataset used
	Optimizer	SGD	Optimizer type
	Epoch	28 - 44	Number of training epochs
	Learning Rate (lr)	0.1	Initial learning rate
	lr Scheduler	Cosine Annealing	Learning rate decay method
	Weight Decay	1e − 4	Regularization strength
	Batch Size per GPU	64	Batch size for single-GPU training
	GPU	1	Number of GPUs used

## 3. Results

This section reports the test results of our modular graph-based models on three datasets: CIFAR-10, CIFAR-100, and MNIST. We demonstrate that using modular graph architectures—developed through the use of sparsified community detection algorithms coupled with PCA-based dimensionality reduction—considerably enhances training efficiency with competitive accuracy.

For all experiments conducted, models were built with ten different communities to give a modular foundation. This controlled connectivity and sparsity along with modularization led to faster convergence compared to traditional, densely connected networks. On CIFAR-10, for example, the standard Barabási–Albert (BA) model took 300 epochs to converge to 95.1% accuracy. Our modular variant of BA, in contrast, converged to 92.9% accuracy in just 44 epochs, over six times faster convergence.

The same pattern was witnessed on CIFAR-100, where the baseline BA took 300 epochs to achieve 76.3%, while the modular one gained 72.9% in just 47 epochs.

The performance gain was especially pronounced on the MNIST dataset. Our Small-World and KMeans modular graph topologies achieved 99.57% and 99.5% accuracy, respectively, in under 30 epochs. As far as we comprehend, these accuracies are the best reported so far for MNIST in spiking neural network (SNN) topologies. These findings demonstrate the effectiveness of modular topologies in leveraging low complexity and spatial redundancy of simpler datasets.

One prominent observation throughout all datasets is that the quantity of nodes minimally impacted model performance. Rather, edge sparsity and organized connectivity among communities were the prevailing factors for convergence rate and generalization. Although global connectivity was maintained by all models, the enforced modular structures encouraged effective information flow, minimized redundancy, and enabled localized learning within partitions of the graph.

To better measure training efficiency more formally, we define the accuracy per epoch (Acc/Epoch) metric as:


$$Acc/Epoch=\frac{Accuracy(\%)}{Epochs}$$ (17)

This metric reflects a model’s capacity to learn effectively with time and provides a fair comparison across models trained with different epoch budgets. Higher Acc/Epo implies faster convergence and greater efficiency—especially desirable in real-time or constrained-resource applications.

Beyond serving as a quantitative comparison metric, Acc/Epoch also reflects how effectively a given network topology translates structural organization into functional learning behavior. Structurally, modular graphs constrain connectivity through dense intra-community links and sparse inter-community connections, limiting redundant global interactions while preserving essential communication pathways. Functionally, these structural constraints influence spike propagation and optimization dynamics, often facilitating faster convergence without compromising accuracy. In this sense, the efficiency metric provides an indirect indicator of how representational capacity emerges from the interaction between structural organization and learning dynamics.

Tables 3 through 5 present the comparisons between our modular graph models and several cutting edge SNN and NAS-based models on CIFAR-10, CIFAR-100, and MNIST, correspondingly. The tables list key statistics including parameter count, training epochs, accuracy, and Acc/Epoch score.

In CIFAR-10, our KMeans-based model was the most effective with an accuracy of 92.53% within just 39 epochs (Acc/Epoch = 2.37). Other modular platforms like Louvain and Small-World also showed high performance with more than 92% accuracy rates along with training efficiency (Acc/Epoch) levels above 2.0. In contrast, most high-accuracy existing models took between 100 to 600 epochs to reach Acc/Epo scores below 1.0.

The same patterns emerged on CIFAR-100. The KMeans-based model scored 72.83% at 40 epochs (Acc/Epoch = 1.82), outperforming most rivals in training efficiency. The Small-World and Louvain models also ranked high in Acc/Epo rates, all above 1.5. As we can see, while some baseline models such as Che et al. (2022) were slightly better (76.25%), they spent much more time on training and thus had much worse Acc/Epoch values (e.g., 0.763).

On MNIST, the Small-World graph model achieved 99.57% accuracy in 28 epochs (Acc/Epoch = 3.556), the most efficient of all the models and datasets. The KMeans model was a close second with Acc/Epoch = 3.317. These statistics point out that modular graph-based models are not just accurate but also very efficient learners. In brief, our experimental findings corroborate the central postulate of this research: the utilization of modularity in graph topologies—coupled with PCA-based dimensionality reduction techniques—yields significantly successful training outcomes while preserving competitive or superior classification performance. Across all benchmarks, our models consistently manifested superior or equal Acc/Epoch values. This superiority was realized without increasing model depth, elevating parameter counts, or resorting to longer training horizons, thereby underscoring the scalability, generalizability, and practical utility of the methodology advanced Table 3.

**Table 3:** Comparison of classification performance on CIFAR-10.

Method	Architecture	Params	Epochs	Accuracy (%)	Acc/Epoch
Rathi et al. (2020) [31]	VGG-9	32 M	20	90.54	4.527
Yan, Zhou, and Wong (2021) [32]	VGG-like	9 M	100 ~ 150	94.16	~0.628 - 0.942
Deng et al. (2022) [33]	ResNet-19	13 M	300	94.44	0.315
Kim et al. (2022) [12]	Searched Architecture	20 M	300	92.73	0.309
Na et al. (2022) [14]	Searched Architecture	21 M	600	93.15	0.155
Che et al. (2022) [13]	Searched Architecture	14 M	100	95.50	0.955
STTS [9]	TANet-Tiny	7 M	300	95.10	0.317
**Graph-based Models (Proposed)**					
SW	TANet-Tiny	6 M	44	92.8	2.107
KMeans	TANet-Tiny	6 M	39	92.53	2.37
Louvain	TANet-Tiny	6.2 M	44	92.87	2.111

## 4. Discussion and conclusion

We introduce in this article a novel graph-based SNN design framework that integrates modular topologies for improving training efficiency and classification accuracy. We validate via controlled comparison on benchmarking datasets—CIFAR-10, CIFAR-100, and MNIST—that the novel architecture speeds up convergence and outperforms state-of-the-art techniques in terms of accuracy while using fewer training epochs and a smaller model size Tables 4–5.

**Table 4:** Comparison of classification performance on CIFAR-100.

Method	Architecture	Params	Epochs	Accuracy (%)	Acc/Epoch
Rathi et al. (2020) [31]	VGG-11	37 M	20	67.87	3.394
Yan et al. (2021) [32]	VGG-like	9 M	100 ~ 150	71.84	~0.479 - 0.718
Deng et al. (2022) [33]	ResNet-19	13 M	300	74.47	0.248
Kim et al. (2022) [12]	Searched Architecture	21 M	300	73.04	0.243
Na et al. (2022) [14]	Searched Architecture	5 M	600	69.16	0.115
Che et al. (2022) [13]	Searched Architecture	14 M	100	76.25	0.763
STTS [9]	TANet-Tiny	7 M	300	76.33	0.254
**Graph-based Models (Proposed)**					
SW	TANet-Tiny	6 M	47	72.7	1.547
KMeans	TANet-Tiny	6 M	40	72.83	1.82
Louvain	TANet-Tiny	6 M	48	72.5	1.510

**Table 5:** Comparison of classification performance on MNIST.

Method	Architecture	Params	Epochs	Accuracy (%)	Acc/Epoch
Rathi et al. (2020) [31]	VGG-9	32 M	20	98.40	4.920
Deng et al. (2022)	ResNet-19	13 M	300	98.80	0.329
Kim et al. (2022) [12]	Searched Architecture	20 M	300	99.28	0.331
Na et al. (2022) [14]	Searched Architecture	21 M	600	99.38	0.166
Che et al. (2022) [13]	Searched Architecture	14 M	100	99.40	0.994
**Graph-based Models**					
SW	TANet-Tiny	6 M	28	99.57	3.556
KMeans	TANet-Tiny	6 M	30	99.50	3.317
Louvain	TANet-Tiny	6.2 M	44	92.48	2.102

### 4.1 Discussion

The employment of modular architectures represents a significant development in the architecture of Spiking Neural Networks (SNNs). By organizing the network as a collection of interconnected modules, we promote a more efficient communication dynamic among neurons, which translates into better learning. Modular architecture enables specialization within the network, with various modules specializing in the learning of various features or functions. This specialization not only results in greater convergence rates but also in the considerable enhancement of generalization performance. Our empirical findings highlight this efficiency: a Small-World and Kmeans SNN trained to an impressive 99.5% accuracy on MNIST in only 28–30 epochs, illustrating the immense potential of modular networks to achieve high performance with drastically lower computational demands.

This work essentially disproves traditional assumptions in SNN design, particularly regarding the primary role of node number. We establish that, whereas node number had a negligible influence on performance, edge connectivity and structure design played a vital role in enhancing convergence rate and fostering the model’s generalization ability. This significant result provides evidence for a deep paradigm shift: rather than simply scaling up the network, emphasis needs to be placed on creating viable modular connectivity schemes that exploit inherent structural strengths.

Importantly, we notice that our structured modular graphs inherently accommodate higher-order interactions (HOIs), which are essential for depicting intricate spatiotemporal patterns that go beyond just the immediate neuronal connections. Unlike shallow SNNs that tend to rely primarily on pairwise interactions, our structured modular graphs naturally enable multi-hop message passing and hierarchical abstraction mechanisms. This design aligns strongly with recent findings in both spiking and graph-based learning literature.

For instance, Jovanović and Rotter [34] demonstrated that specific graph topologies—particularly tree-like motifs—can systematically influence the emergence of third-order correlations in spiking activity. Their work mathematically links recurrent synaptic motifs to measurable HOIs in spike trains, revealing that such correlations are not random but structurally determined. Our modular graphs, by their structured recurrent topologies, are inherently predisposed to these correlation patterns and thus serve as natural substrates for efficient spike-based computation in SNNs.

This result also strongly supports recent observations in Graph Neural Network (GNN) research [35], particularly those made by Huang et al. [36], which emphasize the necessity of leveraging higher-order topologies via simplicial complexes to capture latent group-level dependencies. Their introduction of the HiGCN model, based on flower-petals Laplacians, not only improves classification performance but also offers a principled method for quantifying multi-node interactions via learnable spectral filters. This aligns with our own findings that show how targeted modular topologies can enhance representational capacity even under strict resource constraints.

Thus, rather than unruly increases in depth or width, targeted application of ordered topologies facilitating increased higher-order information flow is seen as a stronger and more effective means of improving learning in SNNs. Collectively, these findings form the foundation for creating energy-efficient, interpretable, and scalable spiking models and make them strongly amenable to real-time and edge computing applications.

In addition to its theoretical contributions, our novel approach has significant practical implications, particularly for real-time applications in resource-constrained settings. The modular architectures introduced play a crucial role in enabling the realistic deployment of SNNs on edge computing and neuromorphic hardware devices, where computational speed and efficiency are essential prerequisites. Moreover, the enhanced interpretability of modular designs significantly adds to their appeal for applications demanding model decision transparency.

### 4.2 Conclusion

In this paper, we introduced a topology-aware modular design paradigm for spiking neural networks, grounded in concepts from graph theory and community detection. By explicitly embedding structured modular connectivity into SNN architectures, we demonstrated that network topology plays a critical role in learning dynamics, convergence behavior, and classification performance. Our experimental results across benchmark datasets confirm that modular graph-based architectures can achieve competitive or superior accuracy while maintaining efficient training dynamics.

Furthermore, we proposed a topology-aware search framework that enables the exploration of richer graph structures beyond the highly constrained search spaces of prior NAS-based SNN approaches. Empirical analysis showed that a 32-node modular configuration provides a practical balance between representational richness, community-aware structure, computational efficiency, and generalization performance.

Overall, this work highlights modular graph-based organization as a promising architectural direction for SNN design. While the current study focuses on static graph structures, future research may investigate adaptive or dynamically evolving modular topologies, theoretical analysis of topology–performance relationships, and deployment on neuromorphic hardware to further explore energy efficiency and scalability in real-world applications.

## Supporting information

S1 Code Complete implementation package. Python source code including all model architecture (PyTorch), data preprocessing scripts, evaluation metrics.
(ZIP)
